# Supplementary material for: Changes in Iron Status Biomarkers with Advancing Age According to Sex and Menopause: A Population-Based Study
Source: J Clin Med. 2023 Aug 16;12(16):5338. doi: 10.3390/jcm12165338 (PMC10455248; doi:10.3390/jcm12165338)
Supplement: Supplementary file 1 [file jcm-12-05338-s001.zip › jcm-2476093-supplementary.pdf]

Table S1a. Effect Estimates of the Association of Age and Sex with Iron Parameters.

	Iron parameters			
	Transferrin saturation		Serum iron	
Adjusted for age and sex	b (95% CI)	P value*	b (95% CI)	P value*
Intercept	21.49 (19.92 to 23.07)	<0.001	16.05 (15.38 to 16.72)	<0.001
Age (years)	0.03 (0.00 to 0.06)	0.026	–0.02 (–0.03 to –0.01)	0.001
Sex (males)	6.07 (3.86 to 8.28)	<0.001	1.68 (1.39 to 1.97)	<0.001
Age x Sex	–0.05 (–0.09 to –0.01)	0.022	–	–
Full model†				
Intercept	24.05 (21.72 to 26.39)	<0.001	22.61 (17.32 to 27.90)	<0.001
Age (years)	0.08 (0.05 to 0.11)	<0.001	–0.11 (–0.21 to –0.02)	0.023
Sex (males)	6.32 (4.12 to 8.53)	<0.001	1.68 (1.36 to 2.00)	<0.001
C-reactive protein‡	–0.81 (–0.98 to –0.64)	<0.001	0.35 (–0.09 to 0.78)	0.12
Waist circumference	–0.05 (–0.07 to –0.03)	<0.001	–0.09 (–0.14 to –0.03)	0.004
Current smoking	1.06 (0.53 to 1.60)	<0.001	0.68 (0.35 to 1.00)	<0.001
Alcohol consumption	–0.58 (–1.13 to –0.04)	0.037	–0.45 (–0.78 to –0.12)	0.008
Age x Sex	–0.05 (–0.09 to –0.01)	0.020	–	–
Age x C-reactive protein	–	–	–0.02 (–0.02 to –0.01)	<0.001
Age x waist circumference	–	–	0.00 (0.00 to 0.00)	0.011

Abbreviation: CI, confidence interval. *Statistical test: linear regression. †Full model adjusted for c-reactive protein, waist circumference, smoking and alcohol. ‡C-reactive protein was binary log-transformed.

Table S1b. Effect Estimates of the Association of Age and Sex with Iron Parameters.

	Iron parameters					
	Ferritin[‡]		Hepcidin[‡]		Soluble transferrin receptor[‡]	
Adjusted for age and sex	b (95% CI)	P value[*]	b (95% CI)	P value[*]	b (95% CI)	P value[*]
Intercept	4.72 (4.62 to 4.82)	<0.001	–0.01 (–0.12 to 0.09)	0.78	1.33 (1.28 to 1.37)	<0.001
$N(Age)_1$	2.80 (2.55 to 3.04)	<0.001	2.72 (2.48 to 2.96)	<0.001	–0.06 (–0.17 to 0.04)	0.23
$N(Age)_2$	1.58 (1.43 to 1.73)	<0.001	1.50 (1.35 to 1.65)	<0.001	0.09 (0.03 to 0.16)	0.004
Sex (males)	2.19 (2.04 to 2.34)	<0.001	1.73 (1.58 to 1.87)	<0.001	–0.03 (–0.09 to 0.04)	0.45
$N(Age)_1$ x Sex	–2.32 (–2.67 to –1.97)	<0.001	–2.52 (–2.87 to –2.18)	<0.001	0.08 (–0.07 to 0.23)	0.31
$N(Age)_2$ x Sex	–1.61 (–1.81 to –1.41)	<0.001	–1.45 (–1.65 to –1.25)	<0.001	0.10 (0.02 to 0.19)	0.02
Full model[†]						
Intercept	4.83 (4.23 to 5.43)	<0.001	–0.40 (–0.68 to –0.13)	0.004	0.91 (0.79 to 1.03)	<0.001
$N(Age)_1$	0.52 (–0.83 to 1.88)	0.45	2.43 (2.19 to 2.67)	<0.001	–0.15 (–0.26 to –0.05)	0.005
$N(Age)_2$	1.34 (0.53 to 2.16)	0.001	1.33 (1.18 to 1.48)	<0.001	–0.03 (–0.10 to 0.03)	0.33
Sex (males)	2.17 (2.00 to 2.33)	<0.001	1.71 (1.56 to 1.86)	<0.001	–0.07 (–0.13 to 0.00)	0.049
C-reactive protein[‡]	0.10 (0.08 to 0.12)	<0.001	0.14 (0.12 to 0.16)	<0.001	–0.01 (–0.03 to 0.01)	0.15
Waist circumference	0.00 (–0.01 to 0.01)	0.58	0.01 (0.00 to 0.01)	0.001	0.00 (0.00 to 0.01)	<0.001
Current smoking	0.02 (–0.05 to 0.09)	0.64	0.06 (–0.01 to 0.13)	0.09	–0.14 (–0.17 to –0.11)	<0.001
Alcohol consumption	–0.29 (–0.36 to –0.22)	<0.001	–0.07 (–0.15 to 0.00)	0.042	0.10 (0.07 to 0.13)	<0.001
$N(Age)_1$ x Sex	–2.58 (–2.96 to –2.21)	<0.001	–2.59 (–2.93 to –2.25)	<0.001	0.11 (–0.04 to 0.26)	0.15
$N(Age)_2$ x Sex	–1.61 (–1.81 to –1.40)	<0.001	–1.46 (–1.65 to –1.27)	<0.001	0.13 (0.04 to 0.21)	0.003
N(Age)1 x Waist circumference	0.02 (0.01 to 0.04)	0.006	–		0.05 (0.01 to 0.10)	0.02
N(Age)2 x Waist circumference	0.00 (–0.01 to 0.01)	0.87	–		0.04 (0.01 to 0.06)	0.006

Abbreviation: CI, confidence interval. [*]Statistical test: linear regression. [†]Full model adjusted for c-reactive protein, waist circumference, smoking and alcohol. [‡]Ferritin, hepcidin, sTfR, and C-reactive protein were binary log-transformed.

Table S1c. Effect estimates of the association of age and sex with iron parameters after exclusion of observations for which residuals were ≥2.58 SD below or above the mean residual.

	Iron parameters			
	Transferrin saturation		Serum iron	
Adjusted for age and sex	b (95% CI)	P value[*]	b (95% CI)	P value[*]
Intercept	20.02 (18.64 to 21.40)	<0.001	14.78 (14.19 to 15.37)	<0.001
Age (years)	0.05 (0.03 to 0.08)	<0.001	0.00 (–0.01 to 0.01)	0.52
Sex (males)	6.22 (4.27 to 8.16)	<0.001	1.48 (1.23 to 1.74)	<0.001
Age x Sex	–0.06 (–0.09 to –0.02)	0.002	–	–
Full model[†]				
Intercept	22.14 (20.10 to 24.18)	<0.001	17.10 (12.50 to 21.69)	<0.001
Age (years)	0.10 (0.07 to 0.12)	<0.001	–0.02 (–0.10 to 0.07)	0.69
Sex (males)	6.40 (4.46 to 8.33)	<0.001	1.50 (1.22 to 1.78)	<0.001
C-reactive protein[‡]	–0.75 (–0.90 to –0.61)	<0.001	0.33 (–0.05 to 0.70)	0.08
Waist circumference	–0.04 (–0.06 to –0.02)	<0.001	–0.04 (–0.09 to 0.01)	0.14
Current smoking	1.01 (0.54 to 1.48)	<0.001	0.63 (0.35 to 0.91)	<0.001
Alcohol consumption	–0.50 (–0.98 to –0.02)	0.040	–0.41 (–0.70 to –0.12)	0.005
Age x Sex	–0.06 (–0.09 to –0.02)	0.002	–	–
Age x C-reactive protein	–	–	–0.01 (–0.02 to –0.01)	<0.001
Age x waist circumference	–	–	0.00 (0.00 to 0.00)	0.31

Abbreviation: CI, confidence interval. [*]Statistical test: linear regression. [†]Full model adjusted for c-reactive protein, waist circumference, smoking and alcohol. [‡]C-reactive protein was binary log-transformed.

Table S1d. Effect estimates of the association of age and sex with iron parameters after exclusion of observations for which residuals were ≥2.58 SD below or above the mean residual.

	Iron parameters					
	Ferritin‡		Hepcidin‡		Soluble transferrin receptor‡	
Adjusted for age and sex	b (95% CI)	P value*	b (95% CI)	P value*	b (95% CI)	P value*
Intercept	5.23 (5.15 to 5.31)	<0.001	0.52 (0.44 to 0.59)	<0.001	1.32 (1.29 to 1.35)	<0.001
$N(Age)_1$	1.82 (1.64 to 2.01)	<0.001	1.71 (1.53 to 1.88)	<0.001	–0.05 (–0.13 to 0.02)	0.18
$N(Age)_2$	0.90 (0.78 to 1.01)	<0.001	0.75 (0.64 to 0.86)	<0.001	0.12 (0.07 to 0.16)	<0.001
Sex (males)	1.43 (1.31 to 1.54)	<0.001	0.99 (0.88 to 1.10)	<0.001	0.01 (–0.03 to 0.06)	0.55
$N(Age)_1$ x Sex	–1.80 (–2.08 to –1.53)	<0.001	–1.55 (–1.80 to –1.30)	<0.001	0.02 (–0.09 to 0.13)	0.74
$N(Age)_2$ x Sex	–1.01 (–1.17 to –0.86)	<0.001	–0.74 (–0.89 to –0.60)	<0.001	0.04 (–0.02 to 0.10)	0.17
Full model†						
Intercept	5.25 (4.79 to 5.71)	<0.001	0.15 (–0.05 to 0.35)	0.15	0.99 (0.91 to 1.08)	<0.001
$N(Age)_1$	0.71 (–0.31 to 1.73)	0.17	1.57 (1.40 to 1.75)	<0.001	–0.12 (–0.19 to –0.04)	0.003
$N(Age)_2$	0.98 (0.38 to 1.59)	0.001	0.68 (0.57 to 0.79)	<0.001	0.01 (–0.04 to 0.06)	0.71
Sex (males)	1.18 (1.06 to 1.30)	<0.001	0.98 (0.87 to 1.09)	<0.001	–0.01 (–0.06 to 0.04)	0.62
C-reactive protein‡	0.05 (0.03 to 0.06)	<0.001	0.06 (0.04 to 0.07)	<0.001	0.00 (–0.01 to 0.02)	0.61
Waist circumference	0.00 (0.00 to 0.01)	0.30	0.00 (0.00 to 0.01)	<0.001	0.00 (0.00 to 0.00)	<0.001
Current smoking	0.02 (–0.03 to 0.08)	0.36	0.05 (0.00 to 0.10)	0.07	–0.13 (–0.16 to –0.11)	<0.001
Alcohol consumption	–0.12 (–0.18 to –0.07)	<0.001	–0.05 (–0.10 to 0.00)	0.05	0.09 (0.07 to 0.11)	<0.001
$N(Age)_1$ x Sex	–1.68 (–1.95 to –1.41)	<0.001	–1.61 (–1.85 to –1.36)	<0.001	0.03 (–0.04 to 0.14)	0.53
$N(Age)_2$ x Sex	–0.77 (–0.93 to –0.62)	<0.001	–0.75 (–0.90 to –0.61)	<0.001	0.07 (0.01 to 0.13)	0.03
N(Age)1 x Waist circumference	0.01 (0.00 to 0.02)	0.21	–		0.01 (–0.02 to 0.05)	0.39
N(Age)2 x Waist circumference	0.00 (–0.01 to 0.00)	0.27	–		0.03 (0.01 to 0.05)	0.004

Abbreviation: CI, confidence interval. *Statistical test: linear regression. †Full model adjusted for c-reactive protein, waist circumference, smoking and alcohol. ‡Ferritin, Hepcidin, sTfR, and C-reactive protein were binary log-transformed.

Table S2a. Effect Estimates of the Association of Menopausal Status with Iron Parameters.

	Iron parameters			
	Ferritin[‡]		Hepcidin[‡]	
Adjusted for age and sex	b (95% CI)	P value*	b (95% CI)	P value*
Intercept	5.01 (4.87 to 5.15)	<0.001	0.27 (0.13 to 0.42)	<0.001
Menopausal status				
Perimenopausal	0.26 (–0.53 to 1.05)	0.52	0.29 (–0.51 to 1.10)	0.47
Postmenopausal	1.45 (0.16 to 2.74)	0.027	1.93 (0.62 to 3.25)	0.004
$N(Age)_1$	–1.36 (–3.64 to 0.92)	0.24	–1.91 (–4.24 to 0.41)	0.11
$N(Age)_2$	–2.39 (–6.78 to 1.99)	0.28	–3.26 (–7.72 to 1.21)	0.15
$N(Age)_1$ x Transition	4.10 (1.49 to 6.71)	0.002	4.53 (1.87 to 7.19)	0.001
$N(Age)_2$ x Transition	1.62 (–1.65 to 4.89)	0.33	7.00 (2.20 to 11.81)	0.004
$N(Age)_1$ x Postmenopausal	6.40 (1.68 to 11.12)	0.008	1.14 (–2.19 to 4.47)	0.50
$N(Age)_2$ x Postmenopausal	2.63 (–1.79 to 7.06)	0.24	3.21 (–1.30 to 7.72)	0.16
Full model‡				
Intercept	5.22 (4.30 to 6.14)	<0.001	0.08 (–0.33 to 0.50)	0.69
Menopausal status				
Perimenopausal	0.20 (–0.57 to 0.97)	0.61	0.25 (–0.54 to 1.04)	0.53
Postmenopausal	1.04 (–0.24 to 2.31)	0.11	1.69 (0.40 to 2.99)	0.010
$N(Age)_1$	–2.78 (–5.94 to 0.39)	0.09	–2.31 (–4.60 to –0.02)	0.048
$N(Age)_2$	–4.55 (–9.02 to –0.09)	0.046	–3.63 (–8.02 to 0.76)	0.10
C-reactive protein	0.12 (0.09 to 0.16)	<0.001	0.15 (0.11 to 0.18)	<0.001
Waist circumference	0.00 (–0.01 to 0.01)	0.91	0.00 (0.00 to 0.01)	0.32
Current smoking	0.07 (–0.04 to 0.18)	0.23	0.13 (0.02 to 0.25)	0.020
Alcohol consumption	–0.32 (–0.44 to –0.19)	<0.001	–0.05 (–0.17 to 0.08)	0.46
$N(Age)_1$ x Transition	4.23 (1.67 to 6.79)	0.001	–0.04 (–0.08 to 0.00)	0.046
$N(Age)_2$ x Transition	2.45 (–0.76 to 5.66)	0.008	7.07 (2.35 to 11.79)	0.003
$N(Age)_1$ x Postmenopausal	6.23 (1.60 to 10.87)	0.14	1.72 (–1.56 to 4.99)	0.30
$N(Age)_2$ x Postmenopausal	2.74 (–1.63 to 7.11)	0.22	3.58 (–0.86 to 8.01)	0.11
$N(Age)_1$ x Waist circumference	0.01 (–0.01 to 0.04)	0.33	–	–
$N(Age)_2$ x Waist circumference	0.02 (0.01 to 0.04)	0.003	–	–

Abbreviation: CI, confidence interval. [*]Statistical test: linear regression. [†]Full model adjusted for c-reactive protein, waist circumference, smoking and alcohol. [‡]Ferritin, Hepcidin, and C-reactive protein were binary log-transformed.

Table S2b. Effect Estimates of the Association of Menopausal Status with Iron Parameters.

	Transferrin saturation		Haptoglobin		Serum iron		Soluble transferrin receptor	
Adjusted for age and sex	b (95% CI)	P value*	b (95% CI)	P value*	b (95% CI)	P value*	b (95% CI)	P value*
Intercept	24.45 (21.63 to 27.28)	<0.001	0.97 (0.81 to 1.12)	<0.001	16.59 (14.88 to 18.31)	<0.001	1.10 (0.94 to 1.27)	<0.001
Menopausal status†								
Perimenopausal	–0.05 (–0.11 to 0.02)	0.15	0.00 (–0.07 to 0.06)	0.92	0.68 (–0.02 to 1.39)	0.06	–0.14 (–0.21 to –0.07)	0.001
Postmenopausal	1.65 (0.49 to 2.82)	0.005	0.01 (–0.08 to 0.10)	0.77	0.98 (–0.03 to 1.99)	0.06	–0.14 (–0.24 to –0.05)	0.004
Age (years)	2.85 (1.19 to 4.52)	0.018	0.01 (0.00 to 0.01)	<0.001	–0.04 (–0.08 to 0.00)	0.06	0.01 (0.00 to 0.01)	0.006
Full model‡								
Intercept	28.30 (24.43 to 32.17)	<0.001	0.50 (0.32 to 0.68)	<0.001	30.56 (21.62 to 39.49)	<0.001	–0.48 (–1.33 to 0.37)	0.27
Menopausal status†								
Perimenopausal	1.52 (0.37 to 2.67)	0.009	–0.02 (–0.07 to 0.04)	0.57	0.66 (–0.05 to 1.37)	0.07	–0.13 (–0.19 to –0.06)	<0.001
Postmenopausal	2.60 (0.95 to 4.25)	0.002	–0.02 (–0.10 to 0.06)	0.60	0.88 (–0.13 to 1.88)	0.09	–0.12 (–0.22 to –0.02)	0.015
Age (years)	0.01 (–0.05 to 0.08)	0.68	0.00 (0.00 to 0.01)	0.10	–0.25 (–0.41 to –0.08)	0.042	0.03 (0.01 to 0.04)	0.001
C-reactive protein	–0.62 (–0.89 to –0.36)	<0.001	0.12 (0.10 to 0.13)	<0.001	0.85 (0.13 to 1.58)	0.022	–0.14 (–0.20 to –0.07)	<0.001
Waist circumference	–0.08 (–0.11 to –0.04)	<0.001	0.01 (0.00 to 0.01)	<0.001	–0.18 (–0.28 to –0.08)	<0.001	0.02 (0.01 to 0.03)	<0.001
Current smoking	1.07 (0.24 to 1.90)	0.012	0.23 (0.20 to 0.27)	<0.001	0.73 (0.22 to 1.23)	0.005	–0.15 (–0.19 to –0.10)	<0.001
Alcohol consumption	–0.21 (–1.13 to 0.70)	0.65	0.01 (–0.04 to 0.05)	0.72	–0.29 (–0.85 to 0.27)	0.31	0.11 (0.06 to 0.16)	<0.001
Age x C-reactive protein	–	–	–	–	–0.02 (–0.04 to –0.01)	0.001	0.00 (0.00 to 0.00)	<0.001
Age x Waist circumference	–	–	–	–	0.00 (0.00 to 0.00)	0.003	0.00 (0.00 to 0.00)	0.001

Abbreviation: CI, confidence interval. *Statistical test: linear regression. †Full model adjusted for c-reactive protein, waist circumference, smoking and alcohol. ‡Ferritin, Hepcidin, and C-reactive protein were binary log-transformed.

Table S2c. Effect Estimates of the Association of Menopausal Status with Hepcidin After Exclusion of Observations for which Residuals were ≥2.58 SD Below or Above the Mean Residual.

	Iron parameters			
	Hepcidin		Ferritin	
Adjusted for age and sex	b (95% CI)	P value*	b (95% CI)	P value*
Intercept	0.61 (0.49 to 0.72)	<0.001	5.27 (5.16 to 5.39)	<0.001
Menopausal status†				
Perimenopausal	0.59 (–0.05 to 1.23)	0.07	0.32 (–0.34 to 0.98)	0.34
Postmenopausal	1.67 (0.66 to 2.68)	0.001	0.45 (–0.66 to 1.56)	0.43
$N(Age)_1$	–0.46 (–2.39 to 1.48)	0.64	0.11 (–1.87 to 2.08)	0.92
$N(Age)_2$	–1.23 (–4.93 to 2.47)	0.51	–0.27 (–4.03 to 3.50)	0.89
$N(Age)_1$ x Transition†	1.90 (–0.29 to 4.09)	0.09	1.91 (–0.33 to 4.15)	0.094
$N(Age)_2$ x Transition†	4.38 (0.44 to 8.32)	0.029	1.32 (–1.51 to 4.15)	0.36
$N(Age)_1$ x Postmenopausal†	–0.44 (–3.11 to 2.23)	0.75	3.59 (–0.43 to 7.61)	0.080
$N(Age)_2$ x Postmenopausal†	1.14 (–2.59 to 4.87)	0.55	0.70 (–3.10 to 4.51)	0.72
Full model‡				
Intercept	0.20 (–0.13 to 0.54)	0.23	4.96 (4.21 to 5.71)	<0.001
Menopausal status†				
Perimenopausal	0.58 (–0.05 to 1.22)	0.07	0.14 (–0.44 to 0.73)	0.63
Postmenopausal	1.57 (0.57 to 2.57)	0.002	0.58 (–0.42 to 1.58)	0.25
$N(Age)_1$	–0.71 (–2.63 to 1.20)	0.46	0.50 (–1.98 to 2.98)	0.69
$N(Age)_2$	–1.42 (–5.07 to 2.23)	0.45	–0.48 (–3.98 to 3.02)	0.79
C-reactive protein	0.09 (0.06 to 0.12)	<0.001	0.07 (0.04 to 0.09)	<0.001
Waist circumference	0.00 (0.00 to 0.01)	0.013	0.01 (0.00 to 0.02)	0.099
Current smoking	0.06 (–0.03 to 0.15)	0.20	0.04 (–0.05 to 0.12)	0.40

Alcohol consumption	–0.01 (–0.10 to 0.09)	0.90	–0.16 (–0.25 to –0.07)	0.001
N(Age)$_1$ x Transition†	1.94 (–0.22 to 4.10)	0.08	1.50 (–0.51 to 3.50)	0.14
N(Age)$_2$ x Transition†	–0.17 (–2.80 to 2.46)	0.90	0.44 (–2.09 to 2.97)	0.73
N(Age)$_1$ x Postmenopausal†	4.25 (0.37 to 8.13)	0.03	2.28 (–1.34 to 5.89)	0.22
N(Age)$_2$ x Postmenopausal†	1.27 (–2.41 to 4.95)	0.50	–0.21 (–3.64 to 3.22)	0.90
N(Age)$_1$ x Waist circumference	–	–	0.00 (–0.02 to 0.02)	0.65
N(Age)$_2$ x Waist circumference	–	–	0.01 (0.00 to 0.02)	0.12

Abbreviation: CI, confidence interval. *Statistical test: linear regression. †Full model adjusted for c-reactive protein, waist circumference, smoking and alcohol. ‡Ferritin, Hepcidin, and C-reactive protein were binary log-transformed.

Table S2d. Effect Estimates of the Association of Menopausal Status with Iron Parameters After Exclusion of … Observations for which Residuals were ≥2.58 SD Below or Above the Mean Residual.

	Transferrin saturation		Haptoglobin		Serum iron		Soluble transferrin receptor	
Adjusted for age and sex	b (95% CI)	P value*	b (95% CI)	P value*	b (95% CI)	P value*	b (95% CI)	P value*
Intercept	22.28 (19.94 to 24.62)	<0.001	1.07 (0.94 to 1.19)	<0.001	15.73 (14.20 to 17.27)	<0.001	1.07 (0.95 to 1.20)	<0.001
Menopausal status†								
Perimenopausal	2.66 (1.71 to 3.61)	<0.001	–0.01 (–0.06 to 0.04)	0.74	1.17 (0.54 to 1.80)	<0.001	–0.13 (–0.18 to –0.07)	<0.001
Postmenopausal	3.81 (2.43 to 5.19)	<0.001	0.03 (–0.04 to 0.11)	0.35	1.56 (0.66 to 2.47)	0.001	–0.13 (–0.21 to –0.06)	<0.001
Age (years)	–0.05 (–0.10 to 0.01)	0.10	0.00 (0.00 to 0.01)	0.007	–0.04 (–0.07 to 0.00)	0.043	0.01 (0.00 to 0.01)	<0.001
Full model‡								
Intercept	26.10 (22.91 to 29.29)	<0.001	0.63 (0.48 to 0.78)	<0.001	26.55 (18.57 to 34.53)	<0.001	0.16 (–0.48 to 0.80)	0.62
Menopausal status†								
Perimenopausal	2.58 (1.64 to 3.52)	<0.001	–0.02 (–0.06 to 0.02)	0.37	1.11 (0.48 to 1.74)	0.001	–0.11 (–0.16 to –0.06)	<0.001
Postmenopausal	3.70 (2.33 to 5.06)	<0.001	0.00 (–0.06 to 0.06)	>0.99	1.45 (0.55 to 2.35)	0.002	–0.11 (–0.18 to –0.04)	0.003
Age (years)	0.00 (–0.06 to 0.05)	0.98	0.00 (0.00 to 0.00)	0.19	–0.18 (–0.33 to –0.03)	0.017	0.02 (0.00 to 0.03)	0.014
C-reactive protein	–0.48 (–0.70 to –0.26)	<0.001	0.09 (0.08 to 0.10)	<0.001	0.67 (0.02 to 1.32)	0.045	–0.07 (–0.12 to –0.02)	0.008
Waist circumference	–0.07 (–0.10 to –0.04)	<0.001	0.01 (0.00 to 0.01)	<0.001	–0.14 (–0.23 to –0.05)	0.002	0.01 (0.00 to 0.02)	0.001
Current smoking	0.50 (–0.19 to 1.18)	0.15	0.18 (0.15 to 0.21)	<0.001	0.62 (0.17 to 1.07)	0.008	–0.13 (–0.17 to –0.10)	<0.001
Alcohol consumption	–0.23 (–0.98 to 0.52)	0.55	0.02 (–0.01 to 0.06)	0.18	–0.34 (–0.84 to 0.16)	0.19	0.11 (0.07 to 0.15)	<0.001
Age x C-reactive protein	–	–	–	–	–0.02 (–0.03 to –0.01)	0.003	0.00 (0.00 to 0.00)	0.006
Age x Waist circumference	–	–	–	–	0.00 (0.00 to 0.00)	0.013	0.00 (0.00 to 0.00)	0.029

Abbreviation: CI, confidence interval. *Statistical test: linear regression. †Full model adjusted for c-reactive protein, waist circumference, smoking and alcohol. ‡Ferritin, Hepcidin, and C-reactive protein were binary log-transformation.

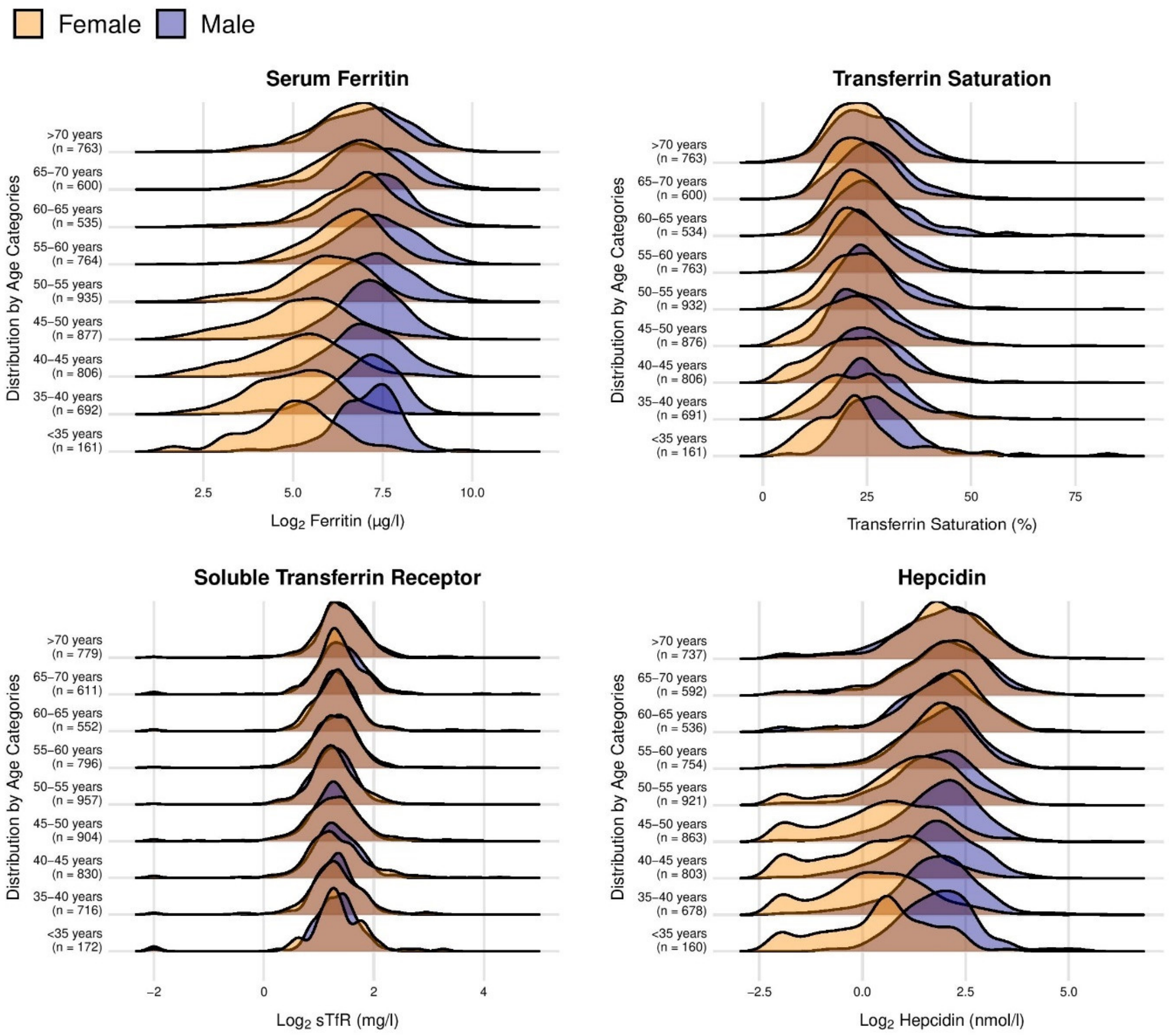

Figure S1. The distribution of different iron measures by sex and age.
